# Ultrasound elastography and 3D volumetry for early postoperative assessment after lymphovenous anastomosis in lower-limb lymphedema

**DOI:** 10.1515/iss-2026-0004

**Published:** 2026-04-28

**Authors:** Sophia-Theresa Diesch, Dmytro Oliinyk, Niklas Biermann, Lukas Prantl, Norbert Heine

**Affiliations:** Department for Plastic, Aesthetic, Hand & Reconstructive Surgery at the University Hospital in Regensburg, Regensburg, Germany; Clinic for Plastic, Aesthetic, Hand and Reconstructive Surgery, Caritas Hospital St. Josef, Regensburg, Germany; Clinic for Plastic, Reconstructive and Aesthetic Surgery, Agaplesion Diakonieklinikum Hamburg GGmbH, Hamburg, Germany

**Keywords:** lymphedema, lower limb, lymphovenous anastomosis, ultrasound elastography, tissue stiffness, objective outcome measurement

## Abstract

**Objectives:**

Lymphovenous anastomosis (LVA) is an established standard of care in the surgical treatment of secondary lymphedema, especially of the lower limbs. However, objective postoperative evaluation remains challenging. Ultrasound elastography offers a quantitative evaluation tool for the assessment of tissue stiffness and may be employed for the detection of subtle postoperative changes in this context.

**Methods:**

12 consecutive patients with primary or secondary lower-limb lymphedema were enrolled in a pilot, prospective observational study with pre- and postoperative elastography and volumetric assessment within the scope of LVA treatment. All measurements were carried out at predefined anatomical regions using a fixed transducer setup to minimise operator bias.

**Results:**

A trend toward postoperatively reduced calf stiffness was observed (−2.31 kPa; p=0.066, *r*=0.54). Additionally, we report significant changes in stiffness symmetry between healthy and affected thighs after LVA surgery (p=0.037, Cohen´s *d* −0.685 [95 %CI −1.105 to −0.040]). Postoperative changes in thigh and knee circumference correlated with elastography parameters (ρ=0.625–0.741; p≤0.030), while volumetric differences were not statistically significant.

**Conclusions:**

Ultrasound elastography may offer an adjunct for the detection of early, subclinical tissue remodelling following LVA surgery. Given its pilot study nature, these findings suggest a potential role for elastography as a complement to conventional measurements and more objective monitoring of surgical outcomes in lymphatic surgery.

## Introduction

While primary lymphedema is mostly caused by congenital malformations of the lymphatic drainage system, secondary lymphedema of the lower extremities is usually seen following iatrogenic interventions on the lymphatic system or radiotherapy [1]. In addition to conservative decongestive therapy with compression stockings and manual lymphatic drainage, lymphatic surgery has more and more established as a treatment for lymphoedema in recent years [2]. Similarly, as microsurgical lymph node transplantation, lymphovenous anastomosis is used in specialized centers to improve lymphatic drainage [2], 3]. In this technique, subcutaneous lymphatic vessels are made visible by staining with indocyanine green and/or patent blue and anastomosed to superficial veins, thereby creating a bypass treating the disruption of natural lymphatic drainage [4], 5]. Palpation, circumferential measurements, body scan and quality of life questionnaires are used to evaluate the success of the operation and to check the postoperative improvement following lymphatic drainage. Ultrasound elastography, an imaging technique for measuring tissue stiffness, offers a relatively new tool for pre- and postoperative quality control in connection with lymphovenous anastomosis, but the existing literature gives little scientific evidence to date [6], [7], [8], [9]. The tissue stiffness in the subcutaneous area of the treated extremity is measured in a defined ultrasound frame as shear-wave or strain elastography.

The aim of our study was to investigate the efficiency of elastography tissue measurement as an additional outcome quality tool after treatment of secondary lymphedema of the lower extremity using lymphovenous anastomosis.

## Materials and methods

The study was carried out with an Ethical Approval of the Institutional Review Board (IRB) of the University of Regensburg, Germany(approval number: 20–1650_1-101). All patients with clinically confirmed primary or secondary lymphedema of the lower extremities who underwent lymphovenous anastomosis (LVA) between March 2023 and September 2024 were evaluated consequently.

A total of 16 patients met the inclusion criteria and were enrolled in this study at our department for Plastic, Hand and Reconstructive Surgery at the University Hospital in Regensburg, Germany. All patients have initially met the inclusion criteria. These criteria include clinically confirmed primary or secondary lymphedema with a surgical indication for LVA surgery in adult (>18 years old) patients, clear asymmetry between affected and non-affected or successfully treated site, prior history of active lymphatic or decongestive therapy, no active panniculitis at the time of surgery, and no contraindications to general anesthesia. All participants provided written informed consent for participation in the study. Patients were excluded in case of bilateral affection, upon local or systemic infection prior to surgery and inability to undergo general anesthesia. Further, participants were eliminated from the analysis in the case of missing paired data or incomplete follow-up. Four patients were lost to follow-up and were therefore excluded from our analysis. A total of 12 consecutive patients were included in our study. Most of the LVAs were performed at the level close to the knee joint (83.3 % either distal ventral thigh or proximal ventral calf, 16.7 % were performed at the distal calf).

**Figure 1:**
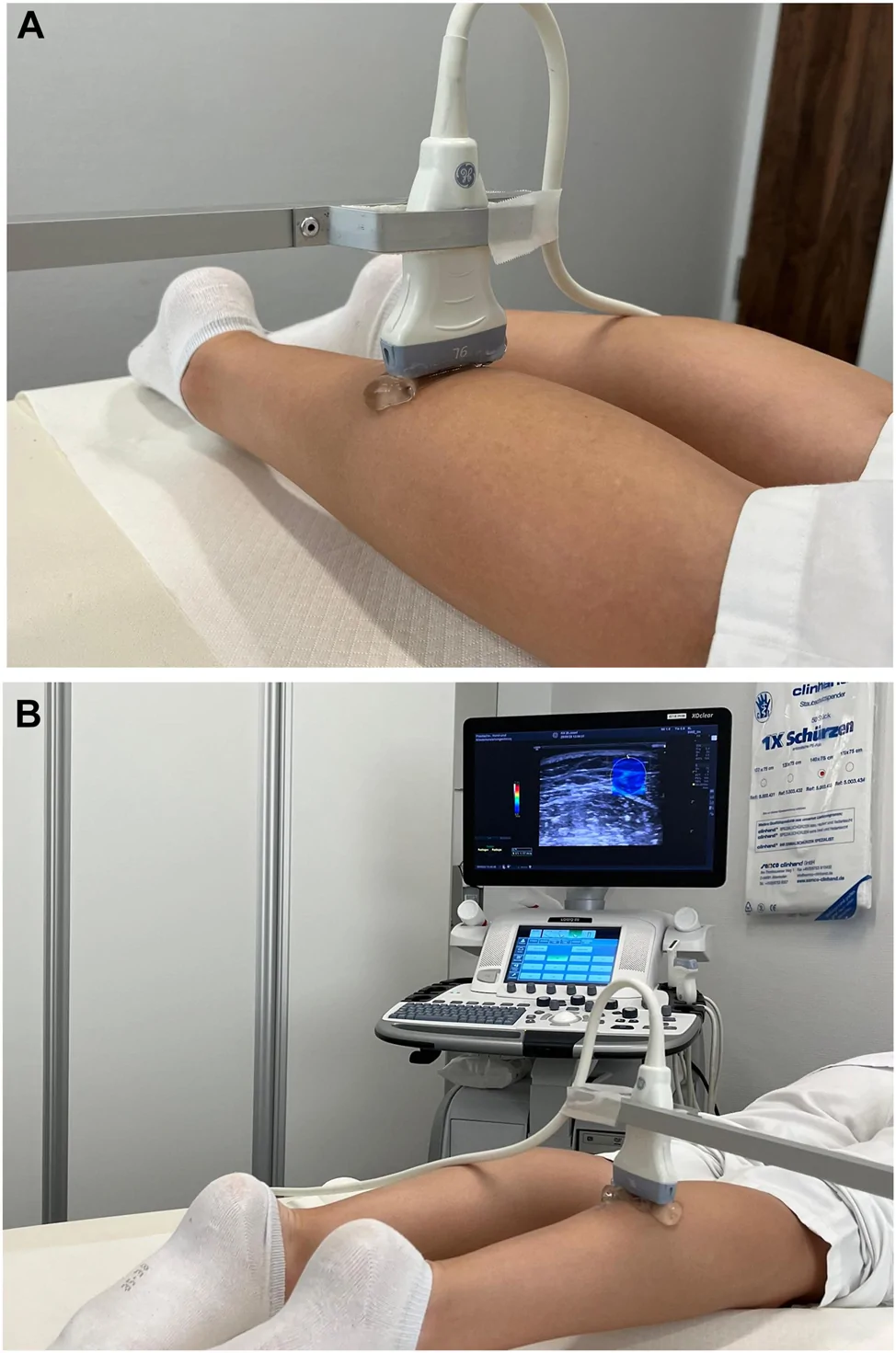
Patients and transducers positioning during elastography. (A – B) exemplary positioning of a patient during elastography assessment in prone position. Standardized placement of the ultrasound transducer at predefined anatomical landmarks (thigh and calf) and after lymphovenous anastomosis (LVA). The fixed orientation of the transducer and minimal compression technique were used to reduce operator-dependent bias.

Ultrasound elastography was utilized to evaluate the biomechanical properties of the soft tissue in both the affected and healthy limbs. The measurements were carried out preoperatively and six weeks postoperatively. For the measurements two standardized anatomical locations per limb were defined. All measurements were carried out twice per location. The locations were defined at 20 cm proximally and 15 cm distally of the knee joint with the patient in the prone position (Figure 1A and B).

To minimize hidden biases through pressure artifacts caused by the operator, the ultrasound transducer (9L transducer, GE LOGIQ E9, GE Healthcare GmbH, trade register HRB 104105, 40468 Düsseldorf, Germany, https://www.gehealthcare.de/) was mounted in a special holder of our own design. A similar arrangement was made for imaging in the “Small Parts” mode. Using this design, the pressure applied to the skin remained constant throughout the examination, thereby not allowing a bias to be introduced by the manipulations of the operator. The depth of measurement was limited to the subcutaneous tissue adjacent to the dermis, within a 1.25 cm region of interest of the studied area. Two elastography measurements were made at each point by recording the speed of the shear wave (m/s) and the elasticity modulus (kPa). To avoid bias, the examination was conducted only by two physicians, S.D. and N.H.

In addition to elastography, circumferential measurements were taken at three specific landmarks:

– 10 cm cranial to the medial knee joint line,
– at the knee joint line,
– 20 cm caudal to the knee joint line.

The circumferential measurements were performed using the 3D volumetric assessments, which were obtained with the Scaneca system (Scaneca GmbH, trade register HRB 202879 B, https://scaneca.de). In this way, the volumetric measurements allowed for a thorough evaluation of limb changes over time. The data was then subsequently analyzed with SPSS Version 29.0.2.0 (20) (IBM, Chicago, IL, USA). The normality of numerical data was pre-analyzed with the Shapiro-Wilk test. For the parametric data *t*-test and Pearson´s correlation was used. Non-parametric data was evaluated using the Wilcoxon Signed-Rank test and Spearman´s rank correlation. Statistical significance was set at p<0.05, and the effects close to significant (p=0.05 to 0.10) were viewed as trends. Effect sizes for parametric tests were calculated using Cohen’s *d* [10]. For non-parametric analyses (Wilcoxon signed-rank test), effect size was expressed as *r* and calculated as $r=Z/\sqrt{N}$ . Sample size estimation for future confirmatory studies was performed using G*Power 3.1 for a paired t-test, assuming α=0.05 and power=0.80 based on the observed effect size (Cohen’s *d*s) [10], 11]. Only significant or near-significant variables were taken into consideration for the further size estimations. Analyses based on non-parametric tests (Wilcoxon signed-rank test) and exploratory correlation analyses were not used for sample size estimation. The Ethics Committee of our tertiary academic care center gave its consent to the study, and all participants signed the informed consent form.

## Results

- Patient characteristics

A total of 12 patients with unilaterally present lymphedema were included into our longitudinal study (Table 1), with a median age of 55 years (range: 41–73). Most of the patients were female (n=9), only three patients were male. The most frequently affected region was the thigh with/without simultaneous affection of the calf (41.4 %), followed by the calf only (33.3 %) and the knee (25 %). Lymphedema predominantly affected the left side (58.3 %) of the patient’s body. The median body mass index (BMI) among our participants was 24.4 kg/m^2^ (range: 18.8–36.1), and the median disease duration was 5.5 years (range: 2–20 years). Secondary lymphedema due to malignancy accounted for 83.3 % of cases. Other sources of lymphedema were primary lymphedema and secondary lymphedema after infection, both of which represented 8.3 % of cases. Classification of lymphedema clinically according to stages indicated that 8.3 % of patients were in stage 1, while 58.3 and 33.3 % were in stage 2 and 3 respectively. Lastly, 75 % of enrolled patients exhibited dents on physical exam by thumb pressure, and 66.7 % had a positive Stemmer sign. The median time from surgery to first follow-up when elastography measurements were carried out was 44 days.

b. Elastography and volumetric changes

**Table 1:** Patients’ characteristics.

Parameter	Value/number
Median age in years	55 (41–73)
Sex	
Male	3 (25 %)
Female	9 (75 %)
Body Mass Index	24.4 (18.8–36.1)
Affected side	
Thigh	5 (41.4 %)
Knee	3 (25 %)
Calf	4 (33.3 %)
Laterality	
Left	7 (58.3 %)
Right	5 (41.7 %)
Reason for lymphedema	
Primary	1 (8.3 %)
Malignancy	10 (83.3 %)
Infection	1 (8.3 %)
Duration of lymphedema in years	5.5 (2–20)
Lymphedema stage	
1	1 (8.3 %)
2	7 (58.3 %)
3	4 (33.3 %)
Dents on physical exam	
Yes	9 (75 %)
No	3 (25 %)
Stemmer sign	
Yes	8 (66.7 %)
No	4 (33.3 %)
Median time from surgery to follow-up in days	44

**Figure 2:**
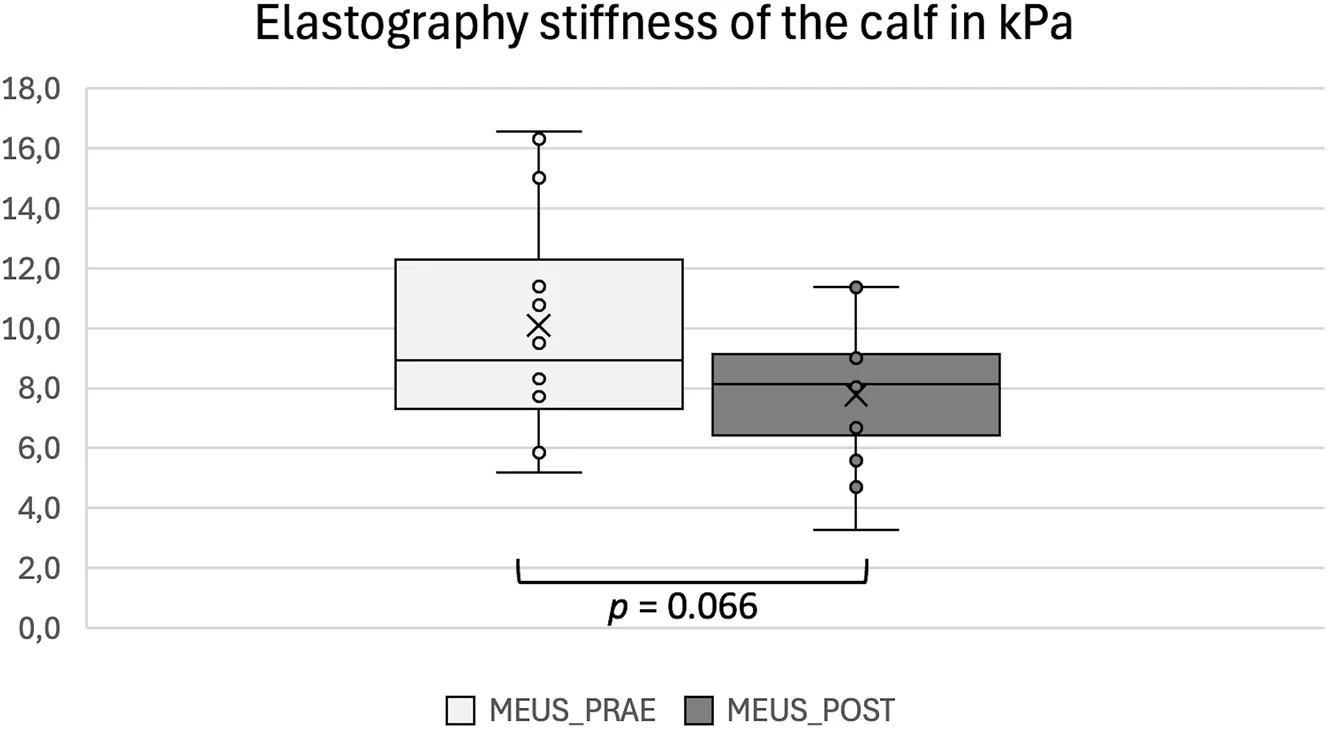
Changes in mean elastography stiffness of the calf in kPa after LVA surgery. Boxplot illustration of paired elastography stiffness measurements of the calf before and after lymphovenous anastomosis. A trend toward reduced postoperative tissue stiffness was observed (p=0.066). Abbreviations: MEUS_PRAE=mean elastography stiffness of the calf on the affected side before surgery, MEUS_POST= mean elastography stiffness of the calf on the affected side after surgery.

Postoperative changes in elastography stiffness and local shear wave velocity were assessed for the thigh and calf. There was no statistically significant difference in elastography stiffness of the thigh (p=0.781) or local shear wave velocity of the thigh (p=0.995, Table 2). However, a trend toward decreased stiffness in the calf was observed (p=0.066, Figure 2). We have also identified a marginal decrease in local shear wave velocity in the calf (p=0.104, Table 2). Postoperatively, a trend towards an increase in thigh circumference was observed (−1.875 cm; p=0.100), as well as an increase in knee circumference (−1.108 cm; p=0.157). The change in calf circumference was not statistically significant (p=0.333). Based on the observed paired effect sizes, an estimated sample size of approximately 23 patients would be required to detect changes in calf elastography stiffness, and approximately 30 patients for local shear wave velocity of the calf, to achieve 80 % statistical power at a two-sided significance level of α=0.05 in future confirmatory paired analyses.

c. Ratio of the healthy side to the affected side

**Table 2:** Parametric paired *t*-test of pre-vs. post-operative values.

Variable	Paired differences			Significance
	Mean difference	Std. error of mean	Cohen´s d with 95 % CI	Two-sided p
Elastography stiffness of the thigh in kPa	0.480000	1.687830	0.082 [95 %CI −0.487 to 0.647]	0.781
Local shear wave velocity of the thigh in m/s	0.000833	0.143471	0.002 [95 %CI −0.564 to 0.567]	0.995
Elastography stiffness of the calf in kPa	2.312500	1.134807	0.588 [95 %CI −0.039 to 1.193]	0.066
Local shear wave velocity of the calf in m/s	0.200417	0.113235	0.511 [95 %CI −0.103 to 1.105]	0.104
Thigh circumference in cm	−1.875000	1.044693	−0.518 [95 %CI −1.113 to 0.097]	0.100
Knee circumference in cm	−1.108333	0.729514	−0.439 [95 %CI −1.024 to 0.164]	0.157
Calf circumference in cm	0.591667	0.583934	0.292 [95 %CI −0.292 to 0.865]	0.333

Volume of the limb and feeling of tension in lymphedema, especially at stadium 1–2, depends on various parameters. As the volume and stiffness of lymphedema-affected extremities may vary depending on daytime and behaviour before the measurements (e.g. several hours in a sitting or standing position during arrival to the examination), the change in difference between the two limbs could be more meaningful than the absolute results. Therefore, the ratio of the healthy side to the affected side was analyzed to evaluate postoperative symmetry as well as to avoid potential hidden biases during follow up given a trend in the increase of limb circumference (Table 2).

**Figure 3:**
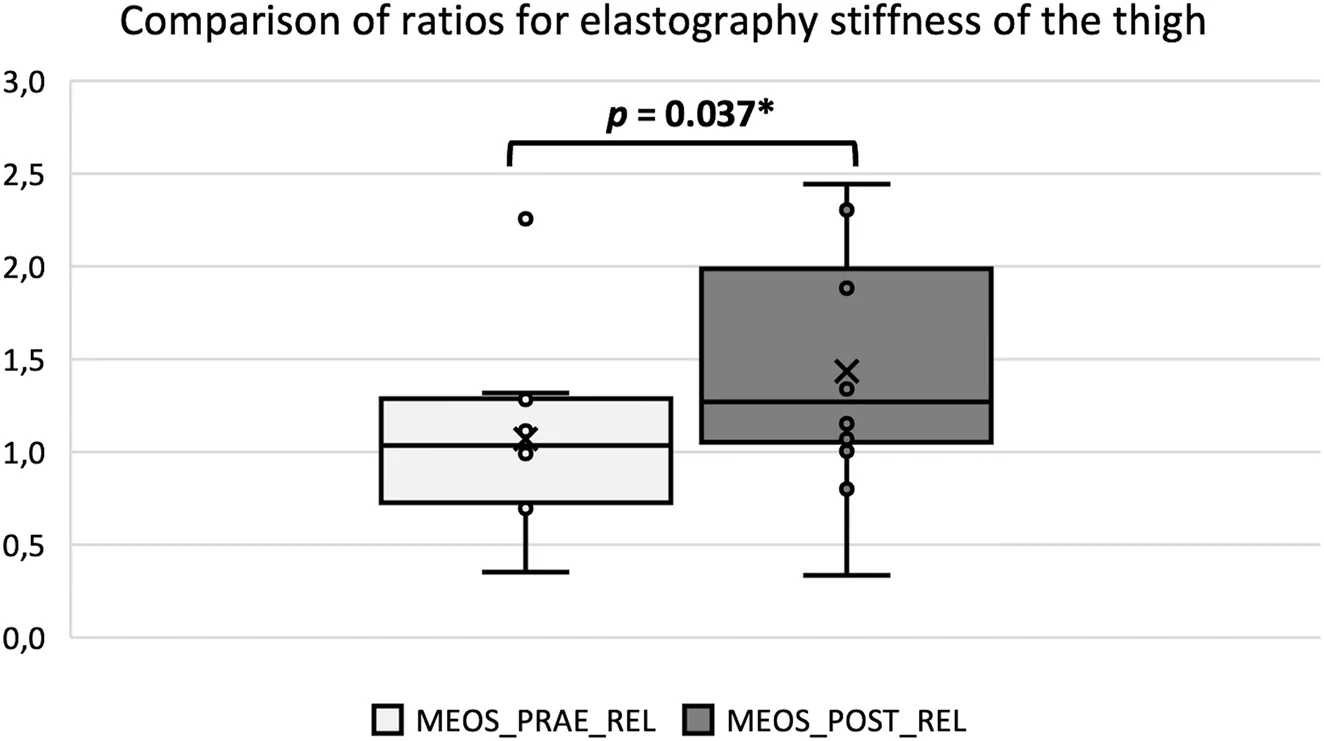
Changes in the ratio of the healthy side to the affected side for elastography stiffness of the thigh. Ratio analysis comparing elastography stiffness between the unaffected contralateral limb and the affected limb before and after surgery. A significant postoperative shift in symmetry between limbs was observed (p=0.037). Abbreviations: MEOS_PRAE_REL=ratio of the mean elastography stiffness of the thigh on the healthy side to the affected side before surgery, MEOS_POST_REL=ratio of the mean elastography stiffness of the thigh on the healthy side to the affected side after surgery, *Statistically significant with p<0.05.

**Figure 4:**
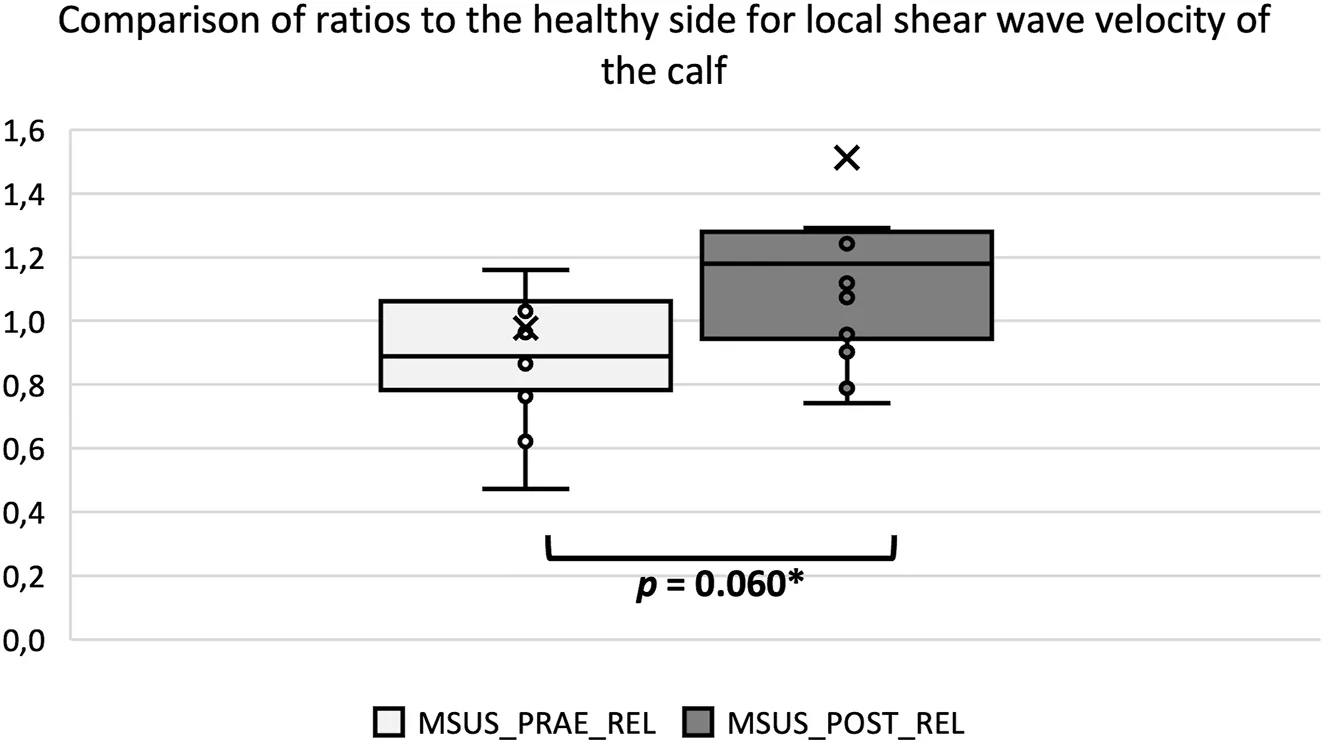
Changes in the ratio of the healthy side to the affected side for local shear wave velocity of the calf. Ratios calculated between the affected and contralateral limb before and after lymphovenous anastomosis. A trend toward normalization of shear wave velocity ratios was observed for both the thigh and calf regions, although without a clear statistical significance. Abbreviations: MSUS_PRAE_REL=ratio of the mean local shear wave velocity of the calf on the healthy side to the affected side before surgery, MSUS_POST_REL=ratio of the mean local shear wave velocity of the calf on the healthy side to the affected side after surgery, *Near-significant trend with p<0.10.

A statistically significant change in the ratio of elastography stiffness of the thigh was observed (p=0.037, Table 3, Figure 3). A trend toward alignment of local shear wave velocity was also observed for the thigh (p=0.138) and the calf (p=0.060, Table 4, Figure 4) after their ratios’ comparison before and after surgery.

**Table 3:** Parametric paired *t*-test of pre-vs. post-operative values: ratio of the healthy side to the affected side.

Variable	Paired differences			Significance
	Mean difference	Std. error of mean	Cohen´s d with 95 % CI	Two-sided p
Ratio of the healthy side to the affected side for elastography stiffness of the thigh	−0.365506	0.154117	**−0.685 [95** **%CI −1.105 to −0.040]**	**0.037** ^**a**^
Ratio of the healthy side to the affected side for local shear wave speed of the thigh	−0.093460	0.058353	−0.462 [95 %CI **−**1.050 to 0.114]	0.138

^a^Statistically significant with p<0.05. Bold values indicate statistically significant results (p<0.05).

**Table 4:** Non-parametric Wilcoxon Signed Ranks test of pre-vs. post-operative values: ratio of the healthy side to the affected side.

Variable	Median difference	Two-sided p-value	Effect size *r*
Ratio of the healthy side to the affected side for elastography stiffness of the calf	0.42	0.136	0.43
Ratio of the healthy side to the affected side for local shear wave velocity of the calf	**0.31**	**0.060** ^**a**^	**0.54**

^a^Statistically significant with p<0.05. Bold values indicate statistically significant results (p<0.05).

d. Correlation analysis

The differences between the changes in the elastography stiffness of the thigh and the changes in the thigh circumference are significantly correlated (Spearman’s Rho=0.625, p=0.030, Table 5). Local shear wave velocity of the thigh also shows a significant correlation with the increase in thigh circumference (Spearman’s Rho=0.741, p=0.006). The delta of the changes in the local shear wave velocity of the calf correlates significantly with those of the knee circumference (Pearson Correlation=0.689, p=0.013). There were no significant correlations for the changes in the calf circumference.

**Table 5:** Parametric and non-parametric correlations before and after LVA surgery.

Variables	Difference in thigh circumference (Spearman’s Rho)	Difference in knee circumference (Pearson)	Difference in calf circumference (Pearson)
Difference in elastography stiffness of the thigh in kPa	**Correlation Coefficient** **0.625** ^**a**^	Correlation Coefficient 0.100	Correlation Coefficient 0.047
	**Significance 0.030** ^**a**^	Significance 0.756	Significance 0.885
Difference in local shear wave speed of the thigh in m/s	**Correlation Coefficient** **0.741** ^**a**^	Correlation Coefficient 0.141	Correlation Coefficient 0.015
	**Significance 0.006** ^**a**^	Significance 0.662	Significance 0.963
Difference in elastography stiffness of the calf in kPa	Correlation Coefficient −0.025	Correlation Coefficient 0.550	Correlation Coefficient 0.371
	Significance 0.937	Significance 0.064	Significance 0.235
Difference in local shear wave speed of the calf in m/s	Correlation Coefficient −0.058	**Correlation Coefficient** **0.689** ^**a**^	Correlation Coefficient 0.463
	Significance 0.858	**Significance 0.013** ^**a**^	Significance 0.129

^a^Statistically significant with p<0.05. Bold values indicate statistically significant results (p<0.05).

## Discussion

We report on a pilot study for utilization of elastography as a diagnostic tool to monitor changes of subcutaneous tissue in a cohort of 12 patients with lymphedema who underwent lymphovenous anastomosis surgery (LVA).

Our study demonstrated a tendency to the decrease in elastography stiffness of the calf by 2.31 kPa (p=0.066) and in local shear wave velocity by 0.20 m/s (p=0.104) following LVA surgery. Additionally, a significant increase in the ratio of elastography stiffness of the healthy to affected side for the thigh was observed (p=0.037), as well as a tendency for the ratio of local shear wave velocity of the calf (p=0.06).

Regarding the efficiency of elastography measurements of the dermis and subcutis, the target areas of lymphedema, De Jong et al. conducted a systematic review in 2017 examining studies of various dermatological conditions and their diagnostic workup using elastography [6]. Particularly for lymphedema, no significant interstage differences in elastography values in a protocol comparing skin and subcutaneous fat tissue of affected individuals and phantoms were reported [6], 8]. In another study evaluating the peak strain force in a 7 mm gel standoff no difference between lymphedema stage II (International Society of Lymphology) and non-affected sites within a patient were reported [6], 7]. On the other hand, there was a significantly higher stiffness noted in the study using the first protocol when comparing lipodermatosclerosis and lymphedema stages 0–3, as in this condition a progressive fibrotic changes of subcutaneous adipose tissue are present [8], 12]. These findings are very important, however, not obvious. In lymphedema, inflammation and tissue fibrosis, particularly in the subcutaneous tissue, are expected, especially in later stages, as recent findings suggest a role for CD4+ T cell depletion in disease progression that in turn may contribute to subcutaneous adipose tissue fibrosis [13], 14]. In our study we did not find any significant differences between affected and non-affected sites at the initial presentation using paired t-test for parametric data (results not shown), which may be attributed to our defined region of interest used in elastography, namely the subcutaneous tissue. Other factors may include different stages represented in our analysis as well as patient specific factors as etiology of a particular case of lymphedema, age, BMI etc. In the study Sanderson et al. inconsistent findings for the stiffness of lymphoedema-affected subcutis have also been reported, whereas dermal stiffness was found to be significantly higher in lymphedema patients [15]. Moreover, Sanderson et al. utilized a ratio values (dermal stiffness to subcutaneous adipose tissue) comparing affected and unaffected sites and reported significant differences between them [15]. So, we decided to focus on the question, if elastography is a feasible tool to monitor changes within subcutaneous tissue following a LVA surgery.

In another study, similar scientific setting was used to monitor skin thickness and tissue elastic modulus induced by intensive decongestive therapy (IDT) in patients with lower limb lymphedema [16]. The study found that IDT reduced subcutis thickness and increased elastic modulus, implying greater tissue stiffness probably due to fluid evacuation [16]. In contrast, in our study we observed a reduction in subcutaneous tissue stiffness following LVA. This may reflect a different, distinct mechanism of action of LVA surgery compared to decongestive therapy. In the study of Lee et al. LVA surgery was shown to be more effective in reducing pressure than volume in lymphedema patients [9]. This aligns with our observations of decreased tissue stiffness post-surgery in the calf. Noteworthy, most of our patients (n=7) had lymphedema at the level of their knee and below, which implies the location of LVA – mostly on the distal thigh. Additionally, we did not observe any significant changes of the limb circumference following LVA surgery (Table 2). Unlike IDT, which is hypothesized to primarily mobilize extracellular fluid to regions with normal lymphatic drainage, LVA directly improves lymphatic drainage by creating functional lymphovenous anastomoses [17]. Therefore, the buildup of interstitial fluid and the pressure in the tissue can be decreased gradually after LVA operation. The reduction in elastography stiffness and shear wave velocity observed in the calf is indicative that LVA is not only effective in alleviating chronic lymphatic overload but also in stopping the development of fibrosis that can eventually result in a softer and more flexible subcutaneous tissue. Nevertheless, the degree to which LVA affects the remodeling of subcutaneous tissue in the long run is still unresolved.

In addition to the decrease in calf stiffness, we observed a significant postoperative increase in the ratio of elastography stiffness between the affected and contralateral healthy sides for the thigh (p=0.037), suggesting ongoing tissue remodelling detectable with elastography. Taking into consideration the location of the LVA (knee joint level) and a trend towards symmetry normalization for the shear wave velocity at the calf level (p=0.060), we hypothesize that early postoperative lymphatic decompression may preferentially influence distal tissue compartments. Therefore, proximal tissue remodelling may occur later. This may be attributed to the role of LVA in the structural changes of the subcutaneous tissue and, hence, in a reduction of fibrotic changes of the affected limb which can be viewed as “tissue normalization” [18], 19]. Most of the currently available studies on lymphedema did not provide a clear answer to the question, what morphological changes are present following LVA surgery. On the macroscopic level, volumetric changes can be monitored using conventional measurements, but also 3-D imaging techniques [20]. A systematic review of Verhey et al. on the objective postoperative changes following LVA surgery could identify 74 studies with a total of 6,260 patients [21]. Their findings indicated an overall average limb volume reduction of 22.67 % after the LVA, while the decrease in excess volume related to lymphedema was measured at 45.52 % with the mean number of anastomoses per patient of 3.9 [21]. Most of the studies have utilized ultrasound sonography as a diagnostic tool within the search for suitable venules or lymphatic vessels. Only the study of Mihara et al. investigated elastography in order to determine the LVA sites in lymphedema, being one of the first studies to integrate this diagnostic tool in the lymphatic surgery [22]. In contrary to their analysis, we focused on the tissue stiffness measured by elastography as a monitoring parameter of the therapy success being the first study group to do so. Even though we did not report any significant circumference changes following LVA, our correlation analysis reveals a significant relationship between elastography stiffness of the thigh and thigh circumference reduction (Spearman’s Rho=0.625, p=0.030) as well as between local shear wave velocity of the thigh and thigh circumference changes (Spearman’s Rho=0.741, p=0.006). Additionally, local shear wave velocity of the calf was significantly correlated with knee circumference reduction (Pearson’s correlation=0.689, p=0.013). Based‍‌‍‍‌ on these results, elastography can be considered a possible biological indicator of the local post-operative changes of subcutaneous tissue in lymphedema. While circumference measurements are still the standard for monitoring clinical outcomes, our findings show that elastography can be used to support these evaluations by identifying alterations in tissue that may lead to a decrease in volume that is measurable from the change in size of the limb. Hence, this is a method that in general can be used as an instrument for objective measurements in lymphatic surgery and may be able to generate more solid scientific ‍‌‍‍‌evidence.

Our study has several limitations, such as a varying duration of the disease and BMI, different stages and etiology of lymphedema and a relatively small number of participants. Despite that our study remains by our knowledge the first one evaluating elastography in the context of monitoring of subclinical changes in lymphedema following LVA surgery. Future research including at least 30 participants should focus on the refinement of available elastography protocols and investigations of long-term tissue remodeling following lymphatic surgery.

## Conclusions

In this observational study, we have portrayed ultrasound elastography as an early and noninvasive tool for detecting subclinical changes in tissue properties that arise following lymphovenous anastomosis in lower extremity lymphedema patients. While conventional outcome parameters like limb circumference and volumetry did not exhibit statistically significant changes six weeks postoperatively, elastography measurements suggested a trend for reduced stiffness in subcutaneous tissues in the calf and a statistically significant difference in stiffness symmetry between the unaffected and affected thigh region. Moreover, we have shown significant correlations between elastography parameters and changes in limb circumference, thereby suggesting that tissue elasticity could serve as a functional candidate biomarker of lymphatic decompression and structural remodeling.

Patients suffering from lymphedema are mostly treated conservatively. Lymphatic surgery like lymphovenous anastomosis as an alternative treatment option for these patients is considered a relatively new technique. All the more we need standardized and reproducible outcome measurement techniques to prove the result and successful treatment of these invasive techniques. In our study, we tried to demonstrate that elastography might provide such a tool, assisting in postoperative follow-up of lymphatic microsurgery. Our findings bode well for the integration of elastography tissue assessments into multimodal monitoring protocols and could eventually enhance the objectivity and sensitivity of outcome evaluation in lymphatic surgery. Larger studies with extended follow-up and standardized elastography protocols are needed to validate these pilot results and further clarify the value of elastography as an adjunct marker for treatment success of disorders involving lymphatics.

## References

[1] Grada AA, Phillips TJ (2017). Lymphedema. J Am Acad Dermatol.

[2] Scaglioni MF, Fontein DBY, Arvanitakis M, Giovanoli P (2017). Systematic review of lymphovenous anastomosis (LVA) for the treatment of lymphedema. Microsurgery.

[3] Gould DJ, Mehrara BJ, Neligan P, Cheng M, Patel KM (2018). Lymph node transplantation for the treatment of lymphedema. J Surg Oncol.

[4] Biermann N, Eschenbacher E, Brébant V, Heine N, Brix E, Prantl L (2024). Patient characteristics may affect the lymphatic staining ability of indocyanine green and patent blue during lymphaticovenous anastomosis. CH.

[5] Chang E, Skoracki R, Chang D (2018). Lymphovenous anastomosis bypass surgery. Semin Plast Surg.

[6] DeJong HM, Abbott S, Zelesco M, Kennedy BF, Ziman MR, Wood FM (2017). The validity and reliability of using ultrasound elastography to measure cutaneous stiffness, a systematic review. Int J Burns Trauma.

[7] Suehiro K, Nakamura K, Morikage N, Murakami M, Yamashita O, Ueda K (2014). Real-time tissue elastography assessment of skin and subcutaneous tissue strains in legs with lymphedema. J Med Ultrasound.

[8] Suehiro K, Morikage N, Murakami M, Yamashita O, Harada T, Ueda K (2015). Skin and subcutaneous tissue strain in legs with lymphedema and lipodermatosclerosis. Ultrasound Med Biol.

[9] Lee J, Kim S, Woo K, Bae H (2022). Effects of lymphovenous anastomosis surgery using ultrasonography in lymphedema from a pressure perspective. Ann Rehabil Med.

[10] Cohen J (2013). Statistical power analysis for the behavioral sciences.

[11] Faul F, Erdfelder E, Lang AG, Buchner A (2007). G*Power 3: a flexible statistical power analysis program for the social, behavioral, and biomedical sciences. Behav Res Methods.

[12] Walsh SN, Santa Cruz DJ (2010). Lipodermatosclerosis: a clinicopathological study of 25 cases. J Am Acad Dermatol.

[13] Rockson SG (2021). Advances in lymphedema. Circ Res.

[14] Zampell JC, Yan A, Elhadad S, Avraham T, Weitman E, Mehrara BJ (2012). CD4^+^ cells regulate fibrosis and lymphangiogenesis in response to lymphatic fluid stasis. PLoS One.

[15] Sanderson J, Tuttle N, Laakso L (2022). Acoustic radiation force impulse elastography assessment of lymphoedema tissue: an insight into tissue stiffness. Cancers.

[16] Zarrad M, Duflos C, Marin G, Benhamou M, Laroche J-P, Dauzat M (2022). Skin layer thickness and shear wave elastography changes induced by intensive decongestive treatment of lower limb lymphedema. Lymphatic Res Biol.

[17] Zaleska M, Olszewski WL, Durlik M (2014). The effectiveness of intermittent pneumatic compression in long-term therapy of lymphedema of lower limbs. Lymphatic Res Biol.

[18] Lee BB, Laredo J, Neville R (2011). Current status of lymphatic reconstructive surgery for chronic lymphedema: it is still an uphill battle. Int J Angiol.

[19] Qiu SS, Pruimboom T, Cornelissen AJM, Schols RM, Van Kuijk SMJ, Van Der Hulst RRWJ (2020). Outcomes following lymphaticovenous anastomosis (LVA) for 100 cases of lymphedema: results over 24-months follow-up. Breast Cancer Res Treat.

[20] Schiltz D, Kiermeier N, Müller K, Diesch ST, Wenzel C, Biermann N (2022). Quality of life evaluation and lack of correlation with volumetric results after lymphovenous anastomoses in lymphedema therapy of the lower extremity. J Vasc Surg Venous Lymph Disord.

[21] Verhey EM, Kandi LA, Lee YS, Morris BE, Casey WJ, Rebecca AM (2022). Outcomes of lymphovenous anastomosis for lower extremity lymphedema: a systematic review. Plastic Reconstr Surg Glob Open.

[22] Mihara M, Hayashi Y, Murai N, Moriguchi H, Iida T, Hara H (2011). Regional diagnosis of lymphoedema and selection of sites for lymphaticovenular anastomosis using elastography. Clin Radiol.

